# Supplementary material for: Genome-Based Reclassification of [Bizionia] algoritergicola Bowman and Nichols 2005 as Algorimicrobium algoritergicola gen. nov., comb. nov. and description of Algorimicrobium bowmanii sp. nov
Source: Microorganisms. 2025 Dec 21;14(1):24. doi: 10.3390/microorganisms14010024 (PMC12843772; doi:10.3390/microorganisms14010024)
Supplement: Supplementary file 1 [file microorganisms-14-00024-s001.zip › microorganisms-4042490-supplementary.pdf]

Genome-based reclassification of [*Bizionia*] *algoritergicola* Bowman and Nichols 2005 as *Algorimicrobium algoritergicola* gen. nov., comb. nov. and description of *Algorimicrobium bowmanii* sp. nov.

Valeriya Kurilenko [1,*], Evgeniya Bystritskaya [1], Nadezhda Otstavnykh [1], Peter Velansky [2], Sergey Baldaev [1], Viacheslav Eremeev [1], Natalya Ageenko [2], Konstantin Kiselev [3], Olga Nedashkovskaya [1] and Marina Isaeva [1,*]

[1] G.B. Elyakov Pacific Institute of Bioorganic Chemistry, Far Eastern Branch, Russian Academy of Sciences, Prospect 100 Let Vladivostoku, 159, Vladivostok 690022, Russia; valerie@piboc.dvo.ru (V.K.); ep.bystritskaya@yandex.ru (E.B.); chernysheva.nadezhda@gmail.com (N.O.); baldaevsergey@gmail.com (S.B.); wieremeew@gmail.com (V.E.); oned2004@mail.ru (O.N.); issaeva@gmail.com (M.I.)

[2] A.V. Zhirmunsky National Scientific Center of Marine Biology, Far Eastern Branch, Russian Academy of Sciences, Palchevskogo Street 17, Vladivostok 690041, Russia; velansky.pv@gmail.com (P.V.); natkuprina@mail.ru (N.A.)

[3] Federal Scientific Center of the East Asia Terrestrial Biodiversity, Far Eastern Branch of the Russian Academy of Sciences, Vladivostok 690022, Russia; kiselev@biosoil.ru (K.K.)

* Correspondence: valerie@piboc.dvo.ru (V.K.); issaeva@gmail.com (M.I.); Tel.: +7-423-231-1168 (V.K.)

Supplementary Materials

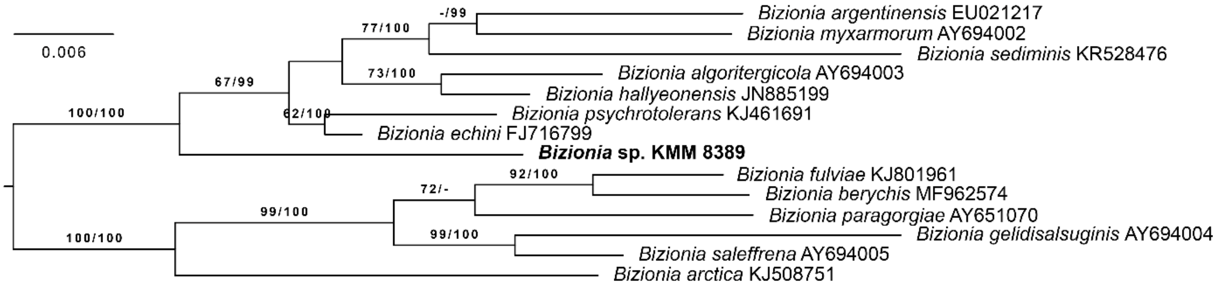

Figure S1. ML/MP 16S rRNA tree showing the position of the new strain KMM 8389^T (in bold) among type strains of the genus *Bizionia*. The ML tree was inferred under the GTR + GAMMA model. The numbers (ML/MP) show bootstrap values greater than 60% measured with 1000 replicates. The bar shows 0.006 substitutions per nucleotide position.

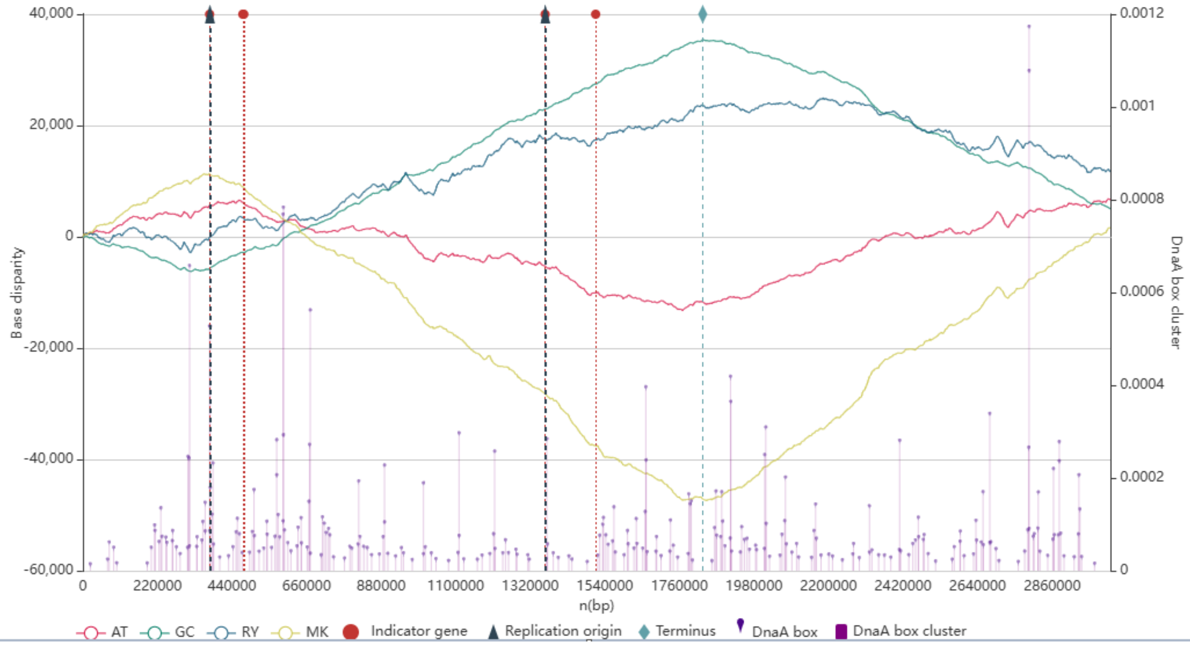

Figure S2. Z-curve figure. The red, green, blue, and yellow line graphs indicate AT (AT-skew), GC (GC-skew), RY (pu-rine/pyrimidine), and MK (amino/keto) disparity. The purple vertical lines indicate the DnaA box cluster. The vertical red, dark blue, and blue dotted lines indicate the locations of indicator genes, replication origins, and terminus, respectively.

Figure S3: Two-dimensional thin-layer chromatograms of polar lipids of strains: (a) KMM 8389[T]; (b) [*B.*] *algoritergicola* CIP 108533[T]; (c) [*B.*] *myxarmorum* CIP 108535[T]; (d) [*B.*] *echini* KMM 6177[T].
Abbreviations: PE, phosphatidylethanoamine; PG, phosphatidylglycerol; PC, phosphatidylcholine; APL, unidentified aminophospholipid; AL1, AL2, unidentified aminolipids; L1-L8, unidentified lipids.

a

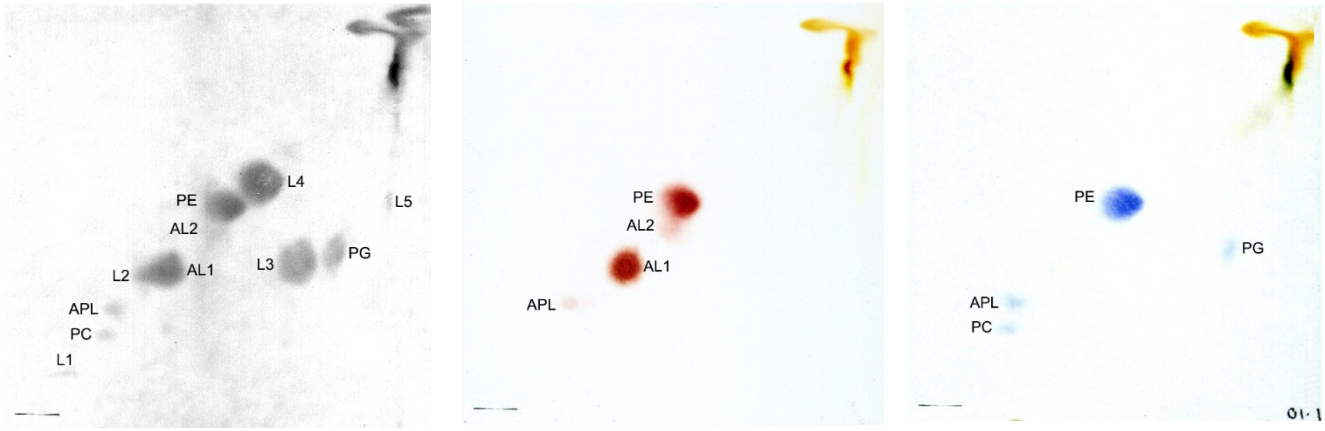

b

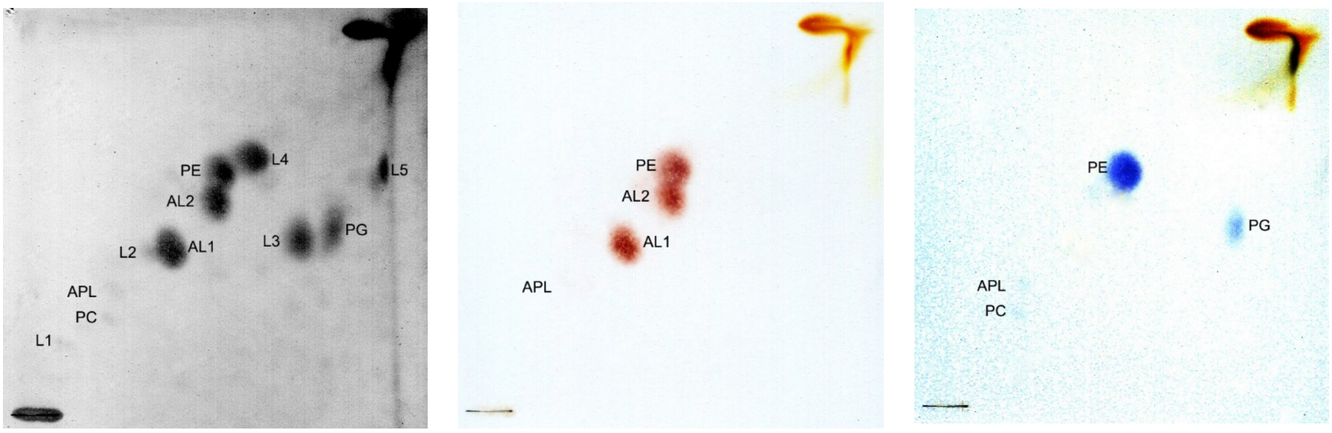

c

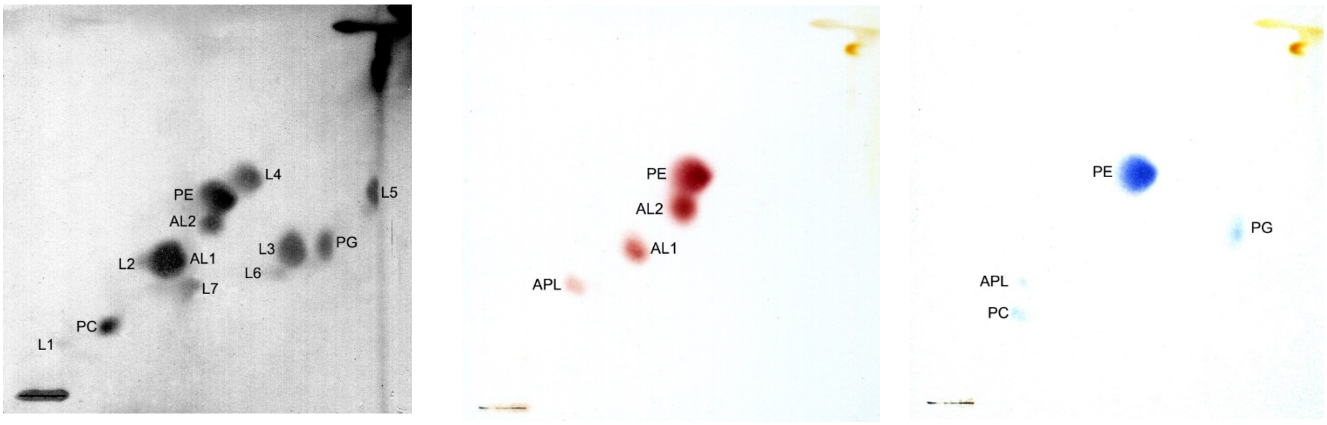

d

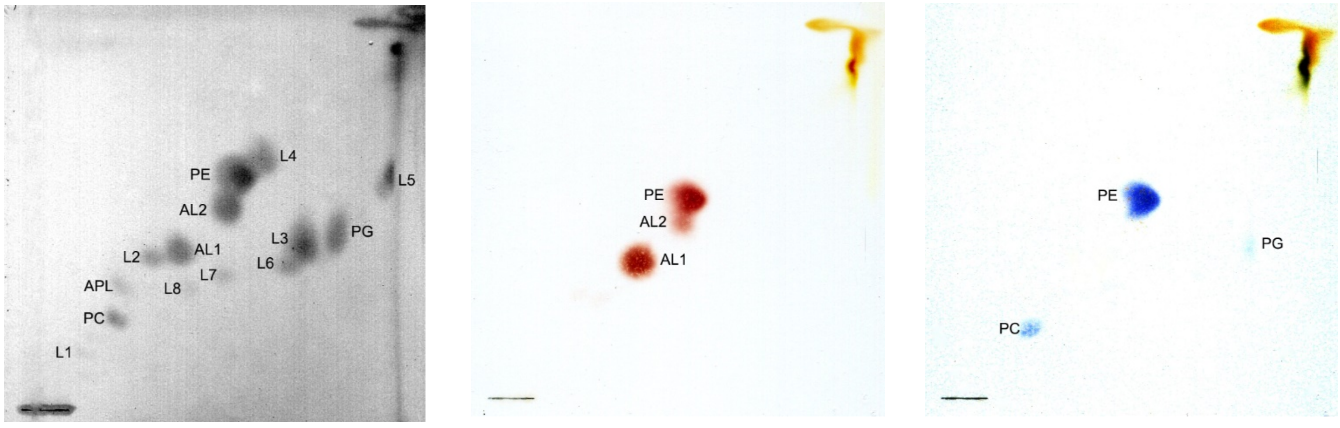

Table S1. Differential physiological characteristics of strains KMM 8389^T, [*B.*] *algoritergicola* CIP 108533^T, [*B.*] *myxarmorum* CIP 108535^T; [*B.*] *echini* KMM 6177^T (data were obtained from the present study).

**Test**	**KMM 8389^T**	[*B.*] *algoritergicola* CIP 108533^T	[*B.*] *myxarmorum* CIP 108535^T	[*B.*] *echini* KMM 6177^T
**API 20NE:**				
Nitrate reduction	-	-	-	-
Indole production	-	-	-	-
Glucose fermentation	-	-	-	-
Arginine dihydrolase	-	-	-	-
Urease	-	-	-	-
Hydrolysis (β- glucosidase) (esculin)	-	-	-	-
Hydrolysis (protease) (gelatin)	-	-	+	+
β-Galactosidase (PNPG)	-	-	-	-
Assimilation of:				
D-glucose	-	-	-	-
L-arabinose	-	-	-	-
D-mannose	-	-	-	-
D-mannitol	-	-	-	-
N-acetylglucosamine	-	-	-	-
D-Maltose	-	-	-	-
D-gluconate	-	-	-	-
Caprate	-	-	-	-
Adipate	-	-	-	-
L-malate	-	-	-	-
Citrate	-	-	-	-
Phenylacetate	-	-	-	-
**API 20E:**				
β-galactosidase (ONPG)	-	-	-	-
Arginine dihydrolase	-	-	-	-
Lysine decarboxylase	-	-	-	-
Ornithine decarboxylase	-	-	-	-
Citrate utilization	-	-	-	-
H_2S production under anaerobic conditions	-	-	-	-
Urease production under anaerobic conditions	-	-	-	-
Tryptophane deaminase	-	-	-	-
Indole production	-	-	-	-
Acetoin production	-	-	-	-
Gelatinase	+	-	+	w
Oxidation of:				
D-glucose	-	-	-	-
D-mannitol	-	-	-	-
Inositol	-	-	-	-
D-sorbitol	-	-	-	-
L-rhamnose	-	-	-	-
D-sucrose	-	-	-	-
D-melibiose	-	-	-	-
Amygdalin	-	-	-	-
L-arabinose	-	-	-	-
**API ZYM:**				
Alkaline phosphatase	+	+	+	+
Esterase (C 4)	+	+	+	+
Esterase Lipase (C 8)	+	+	+	+

Lipase (C 14)	+	+	+	+
Leucine arylamidase	+	+	+	+
Valine arylamidase	+	+	+	+
Cystine arylamidase	+	+	+	+
Trypsin	+	-	-	+
α-chymotrypsin	+	-	-	+
Acid phosphatase	+	+	+	+
Naphtol-AS-BI-phosphohydrolase	+	+	+	+
α-galactosidase	+	-	-	+
β-galactosidase	-	-	-	-
β-glucuronidase	-	-	-	-
α-glucosidase	-	-	-	-
β-glucosidase	-	-	-	-
N-acetyl-β-glucosaminidase	-	-	-	-
α-mannosidase	-	-	-	-
α-fucosidase	-	-	-	-
**Sensitivity to antibiotics:**				
Ampicillin<br>Lincomycin<br>Carbenicillin<br>Vancomycin<br>Cephalexin<br>Rifampicin<br>Erythromycin	+	+	+	+
Kanamycin<br>Nalidixic acid<br>Oxacillin	-	-	-	-
Gentamicin<br>Neomycin	-	-	+	-
Chloramphenicol<br>Streptomycin	+	+	-	+
Cephazolin<br>Tetracycline	-	+	+	+
Polymyxin	-	-	-	+
Doxycycline	-	+	+	-
Ofloxacin	+	+	-	-
Oleandomycin	+	-	+	+
Benzylpenicillin	+	-	+	-

Symbol: (+) – positive, (-) – negative.
